# Evaluation of left ventricular myocardial movement in rats by velocity vector imaging

**DOI:** 10.1371/journal.pone.0239869

**Published:** 2020-10-02

**Authors:** Yuetong Jin, Yihua Gao, Rui Hou, Shanshan Cong

**Affiliations:** 1 Department of Ultrasound, The Affiliated Hospital of Yanbian University, Yanji, Jilin, China; 2 Department of Ultrasound, General Hospital of Tianjin Medical University, Tianjin, Tianjin, China; Nagoya University, JAPAN

## Abstract

**Aim:**

To use velocity vector imaging (VVI) technology to evaluate the correlation between the apical four-chamber view and short-axis myocardial movement in rats.

**Methods:**

We used 25 10-week-old male Sprague-Dawley rats to measure the myocardium peak systolic velocity (Vs; cm/s), peak diastolic velocity (Vd; cm/s), peak systolic strain (SR; %), peak systolic strain rate (SRs; 1/s), and peak diastolic strain rate (SRd; 1/s) from the apical four-chamber view of the left ventricle (LV) and the parasternal mitral valve (PMV)-level short-axis view, and to analyze the correlation between myocardial motion in corresponding views of the two sections.

**Results:**

Comparing the myocardial motion between the lateral wall’s basal segment in the apical four-chamber view of the LV and the lateral wall of the PMV-level short-axis view revealed that the Vd was positively correlated (r = 0.59, p<0.01), as was SRs (r = 0.68, p<0.05). Comparing the myocardial motion between the lateral wall’s middle segment in the apical four-chamber view of the LV and the lateral wall of the PMV-level short-axis view demonstrated that Vd, SRs, and SRd were positively correlated (r = 0.63, 0.82, 0.79, respectively, all p<0.01). Our comparison of myocardial motion between the posterior septum’s basal segment in the apical four-chamber view of the LV and the posterior septum of PMV-level short-axis view showed that Vd and SRs were positively correlated (r = 0.57, 0.68, respectively, both p<0.01). Comparing the myocardial motion between the posterior septum’s middle segment in the apical four-chamber view of the LV and the posterior septum of the PMV-level short-axis view revealed that Vs, Vd, SR, and SRd were positively correlated (r = 0.89, 0.63, 0.64, 0.6, respectively, all p<0.01), and the SRs also had a significant positive correlation (r = 0.53, p<0.05).

**Conclusion:**

VVI technology could be a valuable tool for evaluating the myocardial walls motion of the apical four-chamber view of the rat LV.

## Introduction

Velocity vector imaging (VVI) technology, an ultrasound technology that has progressed recently, is a non-Doppler optical method using speckle tracking that enables the quantification of myocardial movement with frame-by-frame tracking of bright myocardial areas. These natural acoustic markers can be identified on 2D echocardiographic images and tracked by frame to provide velocity and displacement data [1]. The VVI technology computes multiple derivative parameters including the myocardial velocity, strain, and strain rate. At present, VVI technology is used to evaluate the myocardial movement and overall cardiac function in humans and large-animal models in the apical four-chamber view [2]. In rat models, because of the fast heart rate and small heart volume, ultrasound images are often blurred, and due to the difficulties encountered in measurements in different views, most studies of rat models are limited to the short-axis view or the left ventricular apical four-chamber view. We conducted the present study to apply VVI technology to explore the correlation between the apical four-chamber view and the short-axis view, and to obtain more evidence of the myocardial movements in rats.

## Materials and methods

Twenty-five 10-week-old healthy male Sprague-Dawley (SD) rats weighing 180–200 g were purchased from the Yanbian University Experimental Animal Center. The rats were reared for 4 weeks in ventilated cages and constant-temperature rat cages and given a quantitative standard rat feed and unlimited water. The animal experiments were approved by the Animal Experimental Ethics Committee of Yanbian University (Yanji, China) (Protocol No. 2020175). The animal protocols were performed according to the Guide for the Care and Use of Laboratory Animals published by the U.S. National Institutes of Health. We used a Siemens Acuson SC2000 color Doppler ultrasound system which was equipped with VVI technology (Siemens Medical Solutions, Malvern, PA, USA) and a 10V4 probe (frequency 10 MHz) to examine the rats.

**Fig 1:**
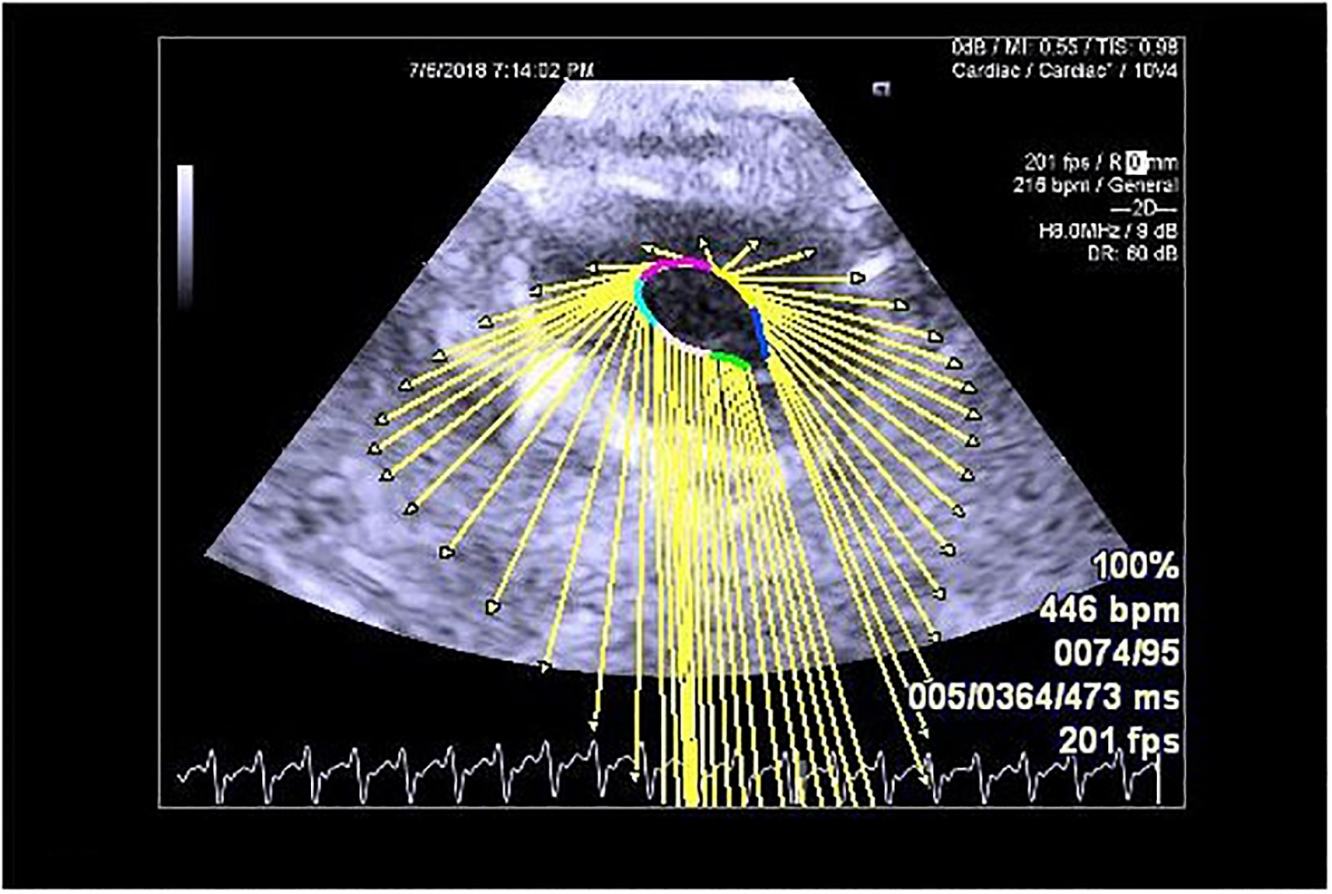
VVI technology to analyze the apical four-chamber view of the left ventricle.

**Fig 2:**
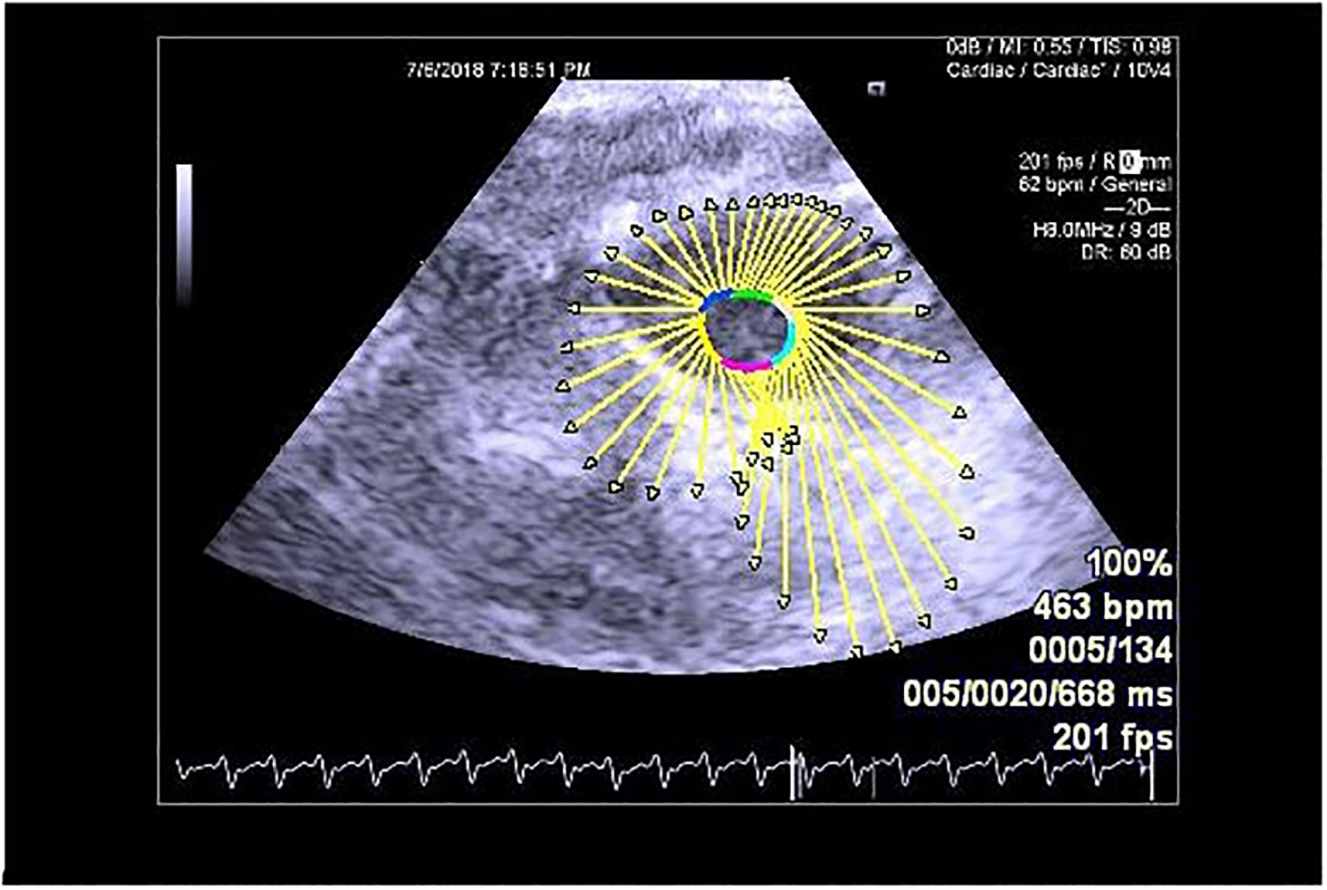
VVI technology to analyze parasternal short axis mitral valve.

At the end of the 4-week protocol, all rats were anesthetized with an intraperitoneal injection of 20% urethane (1.3 mg/kg; Sigma-Aldrich, St. Louis, MO). A physician experienced in making cardiac ultrasound-based diagnoses performed the echocardiography and VVI dynamic image collection on the rats through the apical four-chamber view of the left ventricle and the parasternal mitral valve-level short-axis view. The color Doppler system automatically analyzed the data of each segment, including the lateral wall and posterior septum of the left ventricle of the apical four-chamber view and the lateral wall and posterior septal segment of the parasternal short-axis mitral valve. The parameters of the target segment were collected five times to calculate the average values of the following: the myocardium peak systolic velocity (Vs; cm/s), the peak diastolic velocity (Vd; cm/s), the peak systolic strain (SR; %), the peak systolic strain rate (SRs; 1/s), and the peak diastolic strain rate (SRd; 1/s). We used the absolute values in the comparisons of the measured values, and we conducted a Pearson correlation analysis (Figs 1 and 2). After the analyses, the rats were euthanized by an overdose of urethane (5.0 mg/kg).

## Statistical analyses

The statistical analyses were performed using SPSS 17.0 statistical software (SPSS, Chicago, IL). The measurement data are expressed as the mean±SD, and the correlation analysis was performed by the Pearson method, P-values <0.05 were accepted as significant [3].

## Results

Our comparison of the myocardial motion between the basal segment of the lateral wall of the apical four-chamber view of the left ventricle and the lateral wall of the parasternal mitral valve-level short-axis view revealed that the index Vd was positively correlated (r = 0.59, p<0.01, Table 1), and the index SRs was also positively correlated (r = 0.68, p<0.05). The comparison of myocardial motion between the middle segment of the lateral wall of the apical four-chamber view of the left ventricle and the lateral wall of the parasternal mitral valve-level short-axis view showed that the indexes Vd, SRs, and SRd were positively correlated (r-values 0.63, 0.82, 0.79, respectively, all p<0.01, Table 2).

**Table 1:** Comparison between the basal segment of the lateral wall of the two views.

Part	Vs, cm/s	Vd, cm/s	SR, %	SRs, 1/s	SRd, 1/s
Apical four-chamber view	0.19±0.27	0.21±0.28	2.46±2.88	0.64±0.67	0.62±0.54
Short-axis view	0.33±0.40	0.31±0.31	11.14±11.29	2.98±3.11	3.09±3.01
r-value	0.3	0.68**	0.16	0.59*	0.39
p-value	0.15	0	0.44	0.02	0.06

The correlations between the basal segment of the lateral wall of the apical four-chamber view of the left ventricle and the lateral wall of the parasternal mitral valve-level short-axis view are shown. The data are mean±SD.

*p<0.05.

**p<0.01.

SR: Peak systolic strain, SRd: Peak diastolic strain rate, SRs: Peak systolic strain rate, Vd: Peak diastolic velocity, Vs: Myocardium peak systolic velocity.

**Table 2:** Comparison between the middle segment of the lateral wall of the two views.

Part	Vs, cm/s	Vd, cm/s	SR, %	SRs, 1/s	SRd, 1/s
Apical four-chamber view	0.13±0.17	0.14±0.16	2.15±2.34	0.63±0.59	0.66±0.69
Short-axis view	0.33±0.40	0.31±0.31	11.14±11.29	2.98±3.11	3.01±3.01
r-value	0.24	0.63**	0.24	0.82**	0.79**
p-value	0.25	0.00	0.24	0.00	0.00

The correlations between the middle segment of the lateral wall of the apical four-chamber view of the left ventricle and the lateral wall of the parasternal mitral valve-level short-axis view are shown. The data are mean±SD.

**p<0.01.

Abbreviations are explained in the Table 1 footnote.

The results of our analysis of myocardial motion between the basal segment of the posterior septum of the apical four-chamber view of the left ventricle and the posterior septum of the parasternal mitral valve-level short-axis view demonstrated that the indexes Vd and SRs were positively correlated (r-values 0.57, 0.68, respectively, both p<0.01, Table 3). The comparison of myocardial motion between the middle segment of the posterior septum of the apical four-chamber view of the left ventricle and the posterior valve-level septum of the parasternal mitral valve-level short-axis view showed that the indexes Vs, Vd, SR, and SRd were positively correlated (r; = 0.89, 0.63, 0.64, 0.6, respectively, all p<0.01), and the index SRs was also found to have a significantly positive correlation (r = 0.53, p<0.05, Table 4).

**Table 3:** Comparison between the basal segment of the posterior septum of the two views.

Part	Vs, cm/s	Vd, cm/s	SR, %	SRs, (1/s)	SRd, 1/s
Apical four-chamber view	0.20±0.22	0.21±0.28	1.78±2.16	0.85±1.27	0.54±0.57
Short-axis view	0.16±0.24	0.19±0.35	7.21±9.05	1.85±2.15	1.83±2.03
r-value	0.12	0.57**	0.24	0.68**	0.10
p-value	0.57	0.00	0.25	0.00	0.62

The correlations between the basal segment of the posterior septum of the apical four-chamber view of the left ventricle and the posterior septum of the parasternal mitral valve-level short-axis view are shown. The data are mean±SD.

**p<0.01.

Abbreviations are explained in the Table 1 footnote.

**Table 4:** Comparison between the middle segment of posterior septum of the two views.

Part	Vs, cm/s	Vd, cm/s	SR, %	SRs, 1/s	SRd, 1/s
Apical four-chamber view	0.07±0.14	1.33±1.52	0.48±0.72	0.41±0.41	0.48±0.79
Short-axis view	0.19±0.35	6.10±7.74	1.73±1.93	1.83±2.03	1.83±2.03
r-value	0.89**	0.63**	0.64**	0.53*	0.6**
p-value	0.00	0.00	0.00	0.01	0.00

This table reports the correlation between the middle segment of posterior septum of the apical four-chamber view of the left ventricle and the posterior septum of parasternal mitral valve-level short-axis view. The data are mean±SD.

*p<0.05,

**p<0.01.

Abbreviations are explained in the Table 1 footnote.

## Discussion

At present, ultrasound is a convenient and non-radiation inspection method that has been widely used in cardiac function inspections. VVI is used as an ultrasound spot imaging technique [4], different with Doppler technology. VVI technology can avoid the influence of overall cardiac motion, peripheral myocardial traction, and angle dependence. It is easily operated, noninvasive, and repeatable [5]. The main observation parameters of VVI technology are the indexes Vs, Vd, SR, SRs, and SRd [6], and it is thus more advantageous than conventional ultrasound and tissue Doppler for the evaluation of cardiac function [7]. VVI technology broadens the path for clinical and scientific research, provides an auxiliary means for the diagnosis and treatment of cardiovascular diseases, and has extremely high application value and broad development prospects [8].

VVI was initially explored for the study of the apical four-chamber views and short-axis views of human and medium- and large-animal models [9]. Rats are commonly used as animal models in the experimental research of cardiovascular diseases. With the continuous development of ultrasound technology, rat models are often used to study a variety of myocardial motions and heart functions more and more widely. However, it is very difficult to conduct a conventional ultrasound examination in rats because of their fast heart rate and small heart volume. Few studies of the apical four-chamber view of a rat model have been reported, and animal-model research has been limited to the short-axis view of the aorta or the long-axis view of the left ventricle [10]. The present study appears to be the first to use VVI technology to explore left ventricular myocardial movement in rats, and we plan to further explore the application of VVI technology in evaluations of left ventricular myocardial movement and left ventricular function in rodents.

The results of our comparison of myocardial motion between the basal segment of the lateral wall of the apical four-chamber view of the left ventricle and the lateral wall of the parasternal mitral valve-level short-axis view revealed that the index Vd was positively correlated (r = 0.59, p<0.01), as was the index SRs (r = 0.68, p<0.05). Our analysis of myocardial motion between the middle segment of lateral wall of the apical four-chamber view of the left ventricle and the lateral wall of the parasternal mitral valve-level short-axis view demonstrated that the indexes Vd, SRs and SRd were positively correlated (r-values 0.63, 0.82, 0.79, respectively, all p<0.01). This is similar to the results reported by Wang Yafe [11] in a clinical study, indicating that the apical four-chamber view of the left ventricular sidewall can be a good assessment of myocardial movement in a rat model.

The results of our comparison of myocardial motion between the basal segment of the posterior septum of the apical four-chamber view of the left ventricle and the posterior septum of the parasternal mitral valve-level short-axis view showed that the indexes Vd and SRs were positively correlated (r-values 0.57, 0.68, respectively, both p<0.01). Lastly, our analysis of myocardial motion between the middle segment of the posterior septum of the apical four-chamber view of the left ventricle and the posterior septum of the parasternal mitral valve-level short-axis view showed that the indexes Vs, Vd, SR, and SRd were positively correlated (r = 0.89, 0.63, 0.64, 0.6, respectively, all p<0.01), and the index SRs also had a significant positive correlation (r = 0.53, p<0.05). This is similar to the results of an earlier investigation [12], indicating that the apical four-chamber view of the left ventricular septum in rats has a good display effect and can be suitably used for the study of the heart function of various rat models.

We observed that the Vd, SR, SRs, and SRd were well compared in the correlation of the same segment in two different aspects, which is consistent with previous reports [13, 14]. Our results demonstrate that the above parameters can better confirm whether the same segment in two different views have a good correlation, and that there is no correlation between some segments. Since the short-axis view is easier to manipulate, a large number of references [15, 16] have proven the role of the short-axis view in myocardial motion. This study confirmed the correlation of the two views, and came to the innovative view that the apical four-chamber view can also be used to evaluate the myocardial movement and cardiac function of the left ventricle, and the apical four-chamber view can also observe the heart function of other heart chambers. Therefore, the four-chamber view of the heart will play an important role in the study of the heart in the future. Due to the small heart size and fast heart rate of rats, the visual representation achieve is not as good as those obtained for humans and large animals, and the limited number of experimental samples in this study provides certain limitations regarding the experimental results. In the future, we will further increase the number of animals to overcome these difficulties.

## Conclusions

In summary, our present results demonstrated that it is feasible to use the VVI technique to study the left ventricular myocardial motion and cardiac function in the apical four-chamber view of the rat heart. VVI technology can be used to explore the characteristics of left ventricular segment movement in normal rats, and it provides a basis for experimental research on the movement and cardiac function of various segments of the heart and myocardium in rats.

## Supporting information

S1 File (ZIP)
