# Supplementary material for: Evaluation of left ventricular myocardial movement in rats by velocity vector imaging
Source: PLoS One. 2020 Oct 2;15(10):e0239869. doi: 10.1371/journal.pone.0239869 (PMC7531783; doi:10.1371/journal.pone.0239869)
Supplement: S1 File — (ZIP) [file pone.0239869.s001.zip › Author information.docx]

Evaluation of left ventricular myocardial movement in rats by velocity vector imaging

**Author information：**

Corresponding author's name：Yihua Gao

address：Department of Ultrasoud, The Affiliated Hospital of Yanbian University,Yanji, Jilin

Tel：0086-15526770917

E-mail：[gyh20021997@163.cm](mailto:gyh20021997@163.cm)

First author's name：Yuetong Jin

address：Department of Ultrasoud, The Affiliated Hospital of Yanbian University,Yanji, Jilin

Tel：0086-15044131675

1. mail：[Yuetongjin1@outlook.com](mailto:Yuetongjin1@outlook.com)

Second author's name: Rui Hou

address：Department of Ultrasoud, General Hospital of Tianjin Medical University, Tianjin, Tianjin, 300000, China

Tel：0086-18202561579

1. mail：[kayhou0709@qq.com](mailto:kayhou0709@qq.com)

Third author's name: Shanshan Cong

address：Department of Ultrasoud, The Affiliated Hospital of Yanbian University,Yanji, Jilin

Tel：0086-19889733321

E-mail: [554330507@qq.com](mailto:554330507@qq.com)
