# Supplementary figures and images for: Evaluation of left ventricular myocardial movement in rats by velocity vector imaging
Source: PLoS One. 2020 Oct 2;15(10):e0239869. doi: 10.1371/journal.pone.0239869 (PMC7531783; doi:10.1371/journal.pone.0239869)

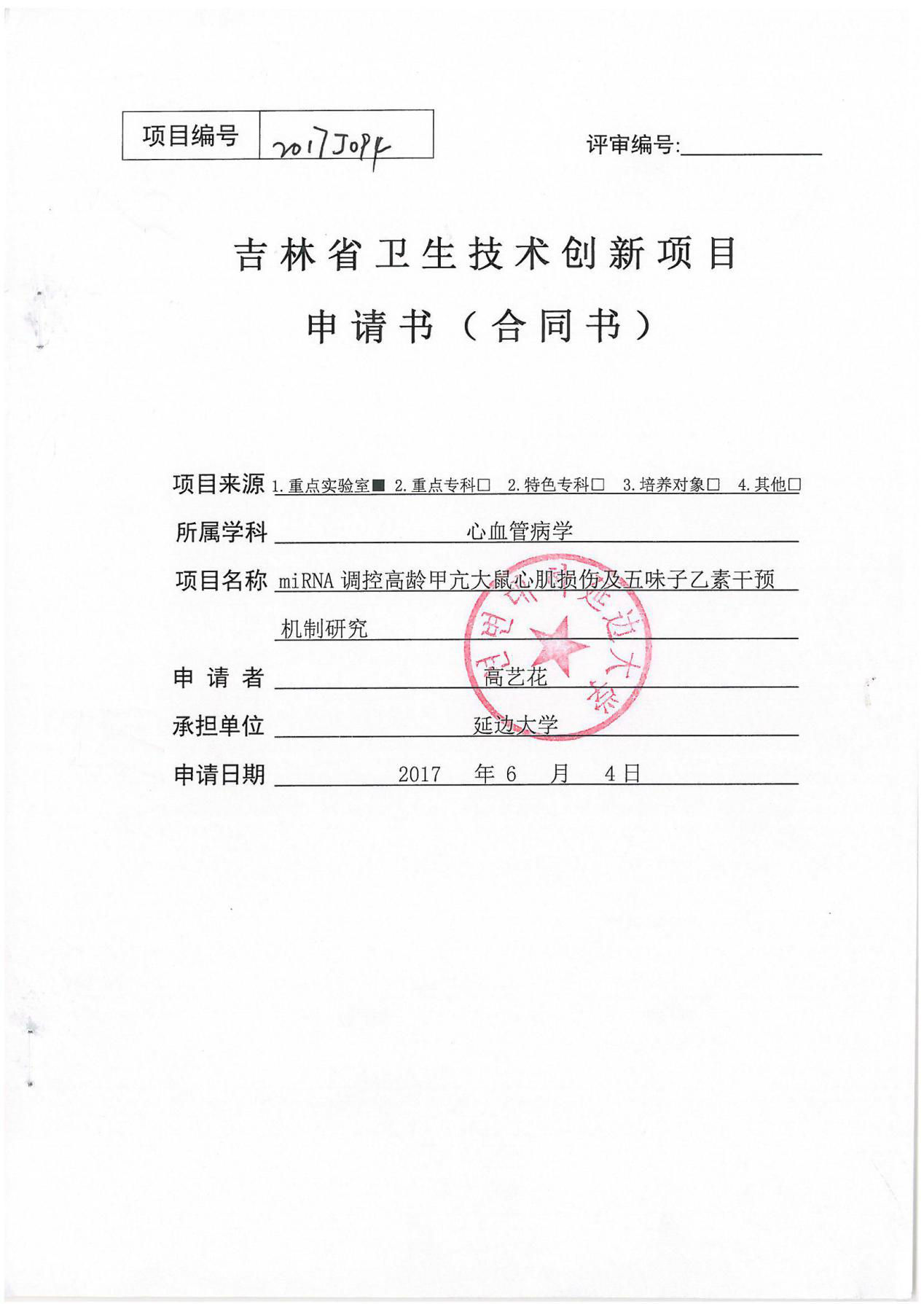

Supplement: S1 File — (ZIP) [file pone.0239869.s001.zip › Jilin Province Health Technology Innovation Project (Number2017J094ú⌐.png]
